# The influence of the Covid-19 pandemic on the 90-day mortality rate after emergency surgery for colon cancer

**DOI:** 10.25122/jml-2022-0108

**Published:** 2022-05

**Authors:** Catalin Vladut Ionut Feier, Calin Muntean, Razvan Bardan, Andra Olariu, Sorin Olariu

**Affiliations:** 1. Department of First General Surgery Clinic, Pius Brinzeu Clinical Emergency Hospital, Timisoara, Romania; 2. Department of Informatics and Medical Biostatistics, Victor Babes University of Medicine and Pharmacy, Timisoara, Romania; 3. Department of Urology, Pius Brinzeu Clinical Emergency Hospital, Timisoara, Romania; 4. Faculty of Medicine, Victor Babes University of Medicine and Pharmacy, Timisoara, Romania

**Keywords:** emergency surgery, 90 days postoperative mortality, colon cancer, Covid-19, severe symptomatology

## Abstract

The Covid-19 pandemic had a significant impact on the treatment of colon cancer. This was due to the redistribution of doctors and medical resources to empower the treatment of Sars-CoV-2-infected patients. Moreover, the restrictions imposed by the authorities on the general population and hospitals were other key elements that had to be taken into consideration. The surgical activity was massively reduced for both elective and emergency surgeries during the pandemic; initially, the elective ones were postponed. This study aimed to analyze the impact of the Covid-19 pandemic on the 90-day postoperative mortality rate of patients who underwent emergency surgery for colon cancer in the First General Surgery Clinic of Pius Brinzeu County Hospital Timisoara. For conducting this study, data from patients who underwent emergency surgery for colon cancer between 26.02.2020–01.10.2021 and the same period of 2016–2017 and 2018–2019 were collected and analyzed, with a p<0.05 being considered statistically significant. As a result, the 90-days postoperative mortality rate increased to 34.5% during the pandemic. A 22.55% rate was observed during 2016–2017 and an 18.4% rate in 2018–2019. In addition, during the pandemic, correlations w ere identified between the presence of 90-day postoperative mortality and severe symptomatology when presenting to the hospital, stage of the disease, and Charlson comorbidity index. All these aspects influenced the 90-days mortality rate of patients undergoing emergency surgery to treat colon cancer during the pandemic.

## INTRODUCTION

The Covid-19 pandemic had an important effect on the activity of surgical clinics around the world. The first person infected with the Sars-CoV-2 virus in Romania was reported on Feb 26, 2020, and it was followed by a national lockdown, introduced on Mar 16, 2020. Among the first steps taken worldwide to cope with the high number of infected patients was increasing the number of beds in intensive care units and redirecting personal and medical resources to provide the care necessary for these patients. Globally, the situation escalated quickly, so all public health systems were overwhelmed by the unpredicted situation [1].

When it comes to the number of surgeries performed during the pandemic, their number significantly decreased. During the first period of the pandemic, elective surgeries were postponed, and only patients who required emergency interventions were able to receive surgical treatment.

This aspect impacted the treatment of patients with colon cancer, this pathology being the third most common form of cancer in men and second in women. Each year approximately 600.000 deaths occur due to this pathology, and the 5-year rate of survival varies between 28% and 60%. This period massively influenced the treatment of colon cancer and its outcome. It is estimated that 2.3 million surgical interventions for colon cancer treatment have been postponed at the height of the pandemic [2, 3].

Along with the restrictions determined by the lockdown, the advice of visiting the hospitals only when severe symptomatology occurs, the fear of patients of contacting the new coronavirus in the hospital, and the decrease in the number of outpatient visits due to epidemiological norms led to an influence on the treatment of colon cancer. Consequently, patients presented in the emergency service with severe symptomatology, an altered biological status, and a more advanced stage of the disease. Thus, the 90 days postoperative mortality was influenced by these parameters and by the limitations of the medical services (their attention mainly being directed to the treatment of Covid-19 patients) [4].

The aim of this study was to investigate the 90-day postoperative mortality rate during the pandemic for patients who underwent emergency surgery for colon cancer treatment and its association with different parameters, all of these compared to the previous periods. The focus was on how the pandemic affected this parameter and not on how the Covid-19 infection could have influenced this outcome.

## MATERIAL AND METHODS

This study included patients who underwent emergency surgery for colon cancer treatment. The patients taken into consideration were treated at the First General Surgery Clinic of the Pius Brinzeu Clinical Emergency County Hospital of Timisoara (this clinic is one of the 3 General Surgery Clinics of the hospital).

The investigated periods were between 26.02.2020 to 01.10.2021 and the same period from the previous years: 2016–2017 and 2018–2019. There were several inclusion criteria for data selection: patients undergoing emergency surgery for colon cancer in the mentioned time intervals, a primary tumor localization varying from cecum to recto-sigmoid junction. During the pandemic, several criteria were added: the patients did not present any specific symptoms for Covid-19 for seven days prior to presentation, lack of a Sars-CoV-2 infection, no history of Covid-19 infection, no Covid-19 infection 90 days postoperative, and a negative RT-PCR result test in the first 24 hours from admission. Regarding the exclusion criteria: patients who underwent elective surgeries outside the mentioned periods, patients with primary tumor located at another level than the one mentioned, patients with Covid-19 at the moment of presentation to the hospital, a history of Covid-19, patients who developed Covid-19 during the 90 days postoperative, and patients with an RT-PCR positive test result.

In order to conduct this study, several parameters of the patients who underwent emergency surgery for colon cancer in the mentioned periods were taken into consideration. We assessed data on year and month of intervention, age, gender, rural or urban origin, presence of 90 days postoperative mortality, presence of severe symptomatology (such as abdominal pain, emesis, reduced flatulence, and hemorrhage of the lower digestive tract), blood transfusions received, postoperative intensive care necessity, presence of other comorbidities (evaluated with the help of Charlson index), the stage of the disease.

The statistical analysis was performed with the IBM SPSS statistic program. The statistical results were considered significant at a p<0.05. For numerical data, central tendency parameters (average, mode, median) and dispersion parameters (standard deviation) were studied. Moreover, statistical tests were applied to show statistically significant differences. Frequency tables were used for categorical data. Chi-square test and correlations between the presence of 90 days of postoperative mortality and different variables from the study were obtained.

## RESULTS

Data from 118 patients who underwent emergency surgery for colon cancer treatment during the pandemic (26.02.2020–01.10.2021) and the same period from 2016–2017 and 2018–2019 were analyzed. The number of patients during the three periods of time is presented in Table 1.

**Table 1:** Distribution of patients during the 3 periods of time.

	2016–2017	2018–2019	2020–2021
**Emergency surgery**	40	49	29

Of the total of 118 patients, 61 were male (51.69%), and 57 were female (48.3%), with an average age of 66.74 (minimum 26 and maximum 97) and a standard deviation of 11.63 years. 77 (65.25%) came from urban areas, and 41 from rural areas (34.75%).

The number of patients who underwent emergency surgery decreased to 40.81% during the pandemic compared to the 2018–2019 period and 27.5% compared to 2016–2017.

The distribution of patients who presented 90 days postoperative mortality during the 3 periods of time is presented in Table 2.

**Table 2:** Distribution of patients with 90 days postoperative mortality.

	2016–2017	2018–2019	2020–2021
**Emergency surgery**	9	9	10

Regarding the entire period of the study, of 118 patients who underwent emergency surgery, 23.7% presented 90 days postoperative mortality. So, in the first period of the study, this rate was 22.5%, in the second period, it was 18.4%, and during the pandemic, the 90 days postoperative mortality increased to 34.5%.

From 2016 to 2017, 37.5% of male patients presented 90-days postoperative mortality, while this rate was 12.5% in women. The second period respected this pattern, so 21.87% of male patients presented 90 days mortality, and only 11.76% of women did so. During the pandemic period, the rate went even higher than before for male (46.15%) and female patients (25%).

When it came to the patients who presented 90 days postoperative mortality, most of them were men throughout the study. So, in 2016–2017 from 9 patients, 66.66% were men; in 2018–2019, 77.77% were men, and this percentage decreased during the pandemic to 60%.

The presence of severe symptomatology in patients who underwent emergency surgery for colon cancer treatment when presenting to the hospital varied during the 3 periods, as shown in Table 3.

**Table 3:** Patients presenting severe symptomatology before undergoing surgery.

	2016–2017	2018–2019	2020–2021
**Emergency surgery**	27 (67.5%)	33 (67.34%)	27 (93.1%)
	p=0.007 between the 3 periods of time		

This parameter was studied in the cases of patients who presented 90-day postoperative mortality, and its variation is presented in Table 4.

**Table 4:** Patients with 90 days mortality and severe symptomatology when presenting to the hospital.

	2016–2017	2018–2019	2020–2021
**Emergency surgery**	5 (55%)	8 (66.6%)	10 (76.92%)

During the pandemic period, the statistical analysis presented a correlation between the presence of severe symptomatology when presenting at the hospital and the presence of 90 days postoperative mortality of those patients (p=0.039).

The number of patients who required blood transfusions postoperative and who presented 90 days postoperative mortality is presented in Table 5.

**Table 5:** Necessity of blood transfusion in patients with 90 days mortality.

	2016–2017	2018–2019	2020–2021
**Emergency surgery**	9 (100%)	6 (50%)	7 (53.38%)

After statistical analysis, a correlation between the presence of 90 days postoperative mortality and the necessity of blood transfusions was obtained in all 3 periods of time with a p<0.05 in all study periods.

Regarding the necessity of intensive care hospitalization and the 90 days postoperative mortality, there was a correlation obtained for all 3 periods of time between these 2 parameters (p<0.001). In the first period of the study, 44% of patients presenting 90 days postoperative mortality required intensive care monitoring; this percentage jumped to 55.55% in the 2018–2019 period and peaked at 77.77% during the pandemic period.

The stage of the disease is an important factor. The distribution of patients who presented 90 days postoperative mortality and the stage of the disease is presented in Table 6.

**Table 6:** The stage of the disease presented by the patients with 90-day mortality.

	2016–2017	2018–2019	2020–2021
**Stage I-II**	3 (33.33%)	2 (22.22%)	0%
**Stage III-IV**	6 (66.66%)	7 (77.77%)	10 (100%)

Over the 3 periods of time, patients with a lower stage of the disease presented lower mortality rates. During the pandemic, 100% of the patients who presented 90 days postoperative mortality had stage III or IV disease (p=0.013).

Regarding the patients' comorbidities (evaluated with the help of the Charlson index), during the pandemic, there was a significant correlation (p<0.001) between the presence of 90 days postoperative mortality and the Charlson index.

## DISCUSSION

In this study, we tried to analyze the impact of the Covid-19 pandemic on 90 days postoperative mortality in patients who underwent emergency surgery for colon cancer during the 26.02.2020–01.10.2021 period and the same period for the years 2016–2017, and 2018–2019.

Of the 118 patients who underwent emergency surgery for colon cancer treatment, 33.89% were treated in the 2016–2017 period, 41.15% in the second period of the study, and 24.57% during the pandemic. This significant decrease in the number of patients during the pandemic period is worth mentioning. In addition, there were 40.81% fewer patients who underwent emergency surgery during the pandemic than in 2018–2019 and 27.5% fewer than in the first period of the study. Such a decrease in acute presentations of patients with colon cancer during the pandemic period was reported in a study in Denmark, where it was also presented a decrease of 48% regarding the emergency surgeries applied for colon cancer treatment [4]. However, after a thorough investigation of different studies from different countries, the 90 days postoperative mortality rate during the pandemic period is very little presented and discussed. The literature presents few studies on the effect of the Covid-19 pandemic on emergency treatment for colon cancer [5–7].

This pandemic had a massive impact on the usual activity of surgical clinics. The indications of visiting the hospitals only when developing severe symptomatology, together with the fear of patients contacting the new coronavirus, led to fewer patients that presented to the hospital. Given that most of the doctors and medical resources were directed to the support and expansion of intensive care capacity, this led to a decrease in the number of surgeries performed [8–12]. There are studies presenting that patients who did not present Covid-19 could undergo surgical procedures for colon cancer treatment during the pandemic, as long as the human and medical resources permit it. However, it must be considered the risk of contacting the Sars-CoV-2 virus in the hospital, but such risk can be significantly reduced by respecting the epidemiological norms [13].

During the entire period of the study, the patients who underwent emergency surgery for colon cancer treatment presented a 23.7% rate of 90 days postoperative mortality. This rate grew from 22.5% during the first period of the study to 34.5% during the pandemic, a growth of almost 50%. When comparing the pandemic rate to the 2018–2019 period, there was almost a doubling in the rate of 90 days mortality, from 18.4% to 34.5%.

This higher mortality rate during the pandemic could also be due to the delay in the diagnosis of the patients, which led to its evolution and severe symptomatology [14, 15]. It is worth mentioning that postponing a surgical treatment more than 6 to 8 weeks must be avoided, because it negatively impacts the patient's survival in the short and long term [16].

In a study from Denmark, the 90 days mortality rate varies from 31% in 2005 to 24% in 2015, these rates being similar to the ones in our study, where there is also a decrease in the 90 days postoperative mortality rate from 22.5% to 18.4%, regarding the periods before the pandemic [17]. In this study, there is no statistically significant difference between the 3 periods; however, it shows an increase of almost 50% from the first period and almost 100% from the 2018–2019 period when it comes to 90 days of postoperative mortality. The lack of statistically significant difference between the pre-pandemic and the pandemic period regarding this aspect is also presented in a study in England [18]. However, this can be explained by the delay in applying epidemiological norms in this country.

The patients who presented severe symptomatology during the 3 periods of time were presented in Table 3. After the statistical analysis resulted in a p=0.007, showing statistically significant differences between the 3 periods of time regarding the presence of severe symptomatology, during the pandemic, only one patient did not present severe symptomatology when presenting to the hospital. This aspect was also mentioned in the literature; during the pandemic, patients presented more severe symptomatology, with studies showing statistically significant differences between the pre-pandemic and the pandemic period (p<0.001) [4]. Moreover, some studies showed an increase of 20.9% in patients who presented severe symptomatology during the pandemic [19].

When it came to patients who presented 90 days postoperative mortality and severe symptomatology when arriving at the hospital, the percentage increased from 55% during the first period to 66.66% during the 2018–2019 period and 76.92% during the pandemic. After the statistical analysis regarding the correlation between the presence of 90-day postoperative mortality and the presence of severe symptomatology, a p=0.039 was obtained for the patients in the pandemic period. The patient's presentation with severe symptomatology is due to patients' fear, postponing the elective interventions (which became emergency ones), and authorities' indications of not visiting the hospitals unless there is more severe symptomatology and the necessity for emergency treatment [8].

Regarding the association between the 90 days postoperative mortality and the necessity of blood transfusions a p<0.001 was obtained, showing a correlation between the two parameters during all three periods of the study. Also, from all the patients that presented 90 days mortality, 78.6% required blood transfusions, whereas, from those who survived more than 90 days, only 31.1% received transfusions. Studies showed that a lower level of pre-operative hemoglobin and a blood transfusion necessity were reported as predictive factors for 1-year postoperative mortality [20]. During the pandemic period, this aspect was justified by postponing surgical acts, delaying the treatment, the increase in severity of symptomatology when presenting to the hospital, and a severe alteration of biological parameters.

The rate of patients presenting 90 days postoperative mortality and needing monitoring in intensive care units was 44.4% during 2016–2017; during the pandemic, this proportion increased by 75%. When it came to comparing 2018–2019 to the 2020–2021 period, an increase of 40% was reported. This shows that more patients needed to be hospitalized in intensive care units during the pandemic due to their advanced pathology, severe symptomatology, the delay in diagnosis and treatment, and the fear of visiting the hospitals.

It is reported that 2.3 million surgeries were postponed during the pandemic period [2, 3]. Most of them were patients who presented an early stage of colon cancer. As presented in Table 5, it can be noticed that during the pandemic, the patients who presented 90-day mortality had stage III or IV. This led to an association between the presence of 90-day postoperative mortality (p=0.013) and the stage of the disease during the pandemic, showing that people presented themselves in a more advanced stage, which influenced their outcome. This predominance of stage III and IV patients during the pandemic was reported in other studies, however, without identifying a significant difference between the two periods from a statistical point of view [4].

This study showed a correlation between 90-days postoperative mortality and the Charlson index during the pandemic (p<0.001). These patients presented an average index of 5.23 during the pandemic. When it came to literature, no statistically significant differences were reported between the periods regarding the Charlson index. However, patients who presented 90 days postoperative mortality had a higher average index than the others [4].

## CONCLUSIONS

The Covid-19 generated pandemic had a massive influence on healthcare systems worldwide. Restrictions imposed by authorities on the population and healthcare systems led to a decrease in the number of emergency surgeries performed for colon cancer treatment during the pandemic.

This study reported an increase in the 90-days postoperative mortality rate in patients who underwent emergency surgery for colon cancer during the pandemic, from 22.5% to 34.5% (compared to the 2016–2017 period) and from 18.4% to 34.5% (compared to the 2018–2019 period).

Due to restrictions and patients' fear, the rate of patients with severe symptomatology when presenting to the hospital was significantly higher during the pandemic than previous periods of time (p=0.007). Moreover, a correlation between the presence of severe symptomatology and 90 days postoperative mortality during the pandemic was obtained (p=0.039).

Regarding the disease stage, all patients who presented 90-days postoperative mortality had stage III or IV of cancer. After applying the statistical tests, a correlation between the two parameters during the pandemic period was obtained (p=0.013). Lastly, during the pandemic, the Charlson index correlated with the 90-days postoperative mortality rate (p<0.001).

The pandemic period had an important impact on the outcome of patients who underwent emergency surgery to treat colon cancer. Since the pandemic reduced the number of surgeries performed, a rise in the number of patients who require treatment for colon cancer is expected in the near future.
